# Interfacial engineering in SnO_2_-embedded graphene anode materials for high performance lithium-ion batteries

**DOI:** 10.1038/s41598-024-67647-w

**Published:** 2024-07-20

**Authors:** Xiaolu Li, Zhongtao Zhao, Yufeng Deng, Dongsheng Ouyang, Xianfeng Yang, Shuguang Chen, Peng Liu

**Affiliations:** School of Materials Science and Engineering, Changsha University of Science and Technology, Changsha, 410114 Hunan People’s Republic of China

**Keywords:** Lithium-ion batteries, Anode materials, Tin dioxide/graphene composites, Interfacial engineering, Materials for energy and catalysis, Energy storage, Energy science and technology, Materials science

## Abstract

Tin dioxide is regarded as an alternative anode material rather than graphite due to its high theoretical specific capacity. Modification with carbon is a typical strategy to mitigate the volume expansion effect of SnO_2_ during the charge process. Strengthening the interface bonding is crucial for improving the electrochemical performance of SnO_2_/C composites. Here, SnO_2_-embedded reduced graphene oxide (rGO) composite with a low graphene content of approximately 5 wt.% was in situ synthesized via a cetyltrimethylammonium bromide (CTAB)-assisted hydrothermal method. The structural integrity of the SnO_2_/rGO composite is significantly improved by optimizing the Sn–O–C electronic structure with CTAB, resulting a reversible capacity of 598 mAh g^−1^ after 200 cycles at a current density of 1 A g^−1^. CTAB-assisted synthesis enhances the rate performance and cyclic stability of tin dioxide/graphene composites, and boosts their application as the anode materials for the next-generation lithium-ion batteries.

## Introduction

There is an urgent need for the high-performance lithium-ion batteries (LIBs) to meet the rapid developments of electric vehicles and smart grids, because the conventional LIBs based on graphite anodes (372 mAh g^−1^) cannot satisfy the growing demand for the efficient energy storage^1,2^. As an alternative anode material, SnO_2_ has received considerable attention due to its low cost, high theoretical specific capacity (1494 mAh g^−1^), high energy density, and excellent safety^3^. However, the SnO_2_ anode undergoes an irreversible conversion reaction during the first charge and discharge process. As a result, some active lithium ions convert to inert substances (Li_2_O, Li_2_CO_3_, LiF etc.), thereby reducing the initial coulomb efficiency (ICE)^4^. When the cut-off voltage is set within the range of 0.01–3.0 V, the ICE ranges only from 43 to 69%^5^. Simultaneously, pulverization and exfoliation of the anode coating caused by the significant volume expansion (~ 260%) during alloying reaction lead to the poor cyclic stability^6^. These bottlenecks limit the commercial application of SnO_2_ anode material for the lithium-ion batteries.

In recent years, a series of strategies have been proposed for improving the electrochemical performance of tin-based anode materials, including nanoscaling^7,8^, modification with carbon materials^9,10^ and design of special structures^11,12^. Modification with graphene is a promising approach due to its inherent structural stability, excellent flexibility, and high electrical conductivity^13^. Firstly, the graphene acts as an exceptional conductor that enables rapid electron transfer, thus enhancing the overall electrical conductivity of the composite materials. Secondly, the cyclic stability of the anode can be improved due to the relieving effect of graphene on the volume expansion of SnO_2_ particles^14^. However, the hydrophobicity of graphene presents challenges in effectively immobilizing the SnO_2_ particles. Although graphene oxide with abundant oxygen-containing functional groups is usually employed to anchor the metal ions, the interfacial adhesion is subject to the agglomeration of SnO_2_ particles. The insufficient interface results in discontinuous conductive channels for the lithium ions and electrons, and weakens the buffering role of graphene as a substrate. Consequently, well-designed tin dioxide/graphene composites should possess uniform particle dispersion, high ionic/electronic conductivity, as well as strong interfacial bonding.

**Figure 1:**
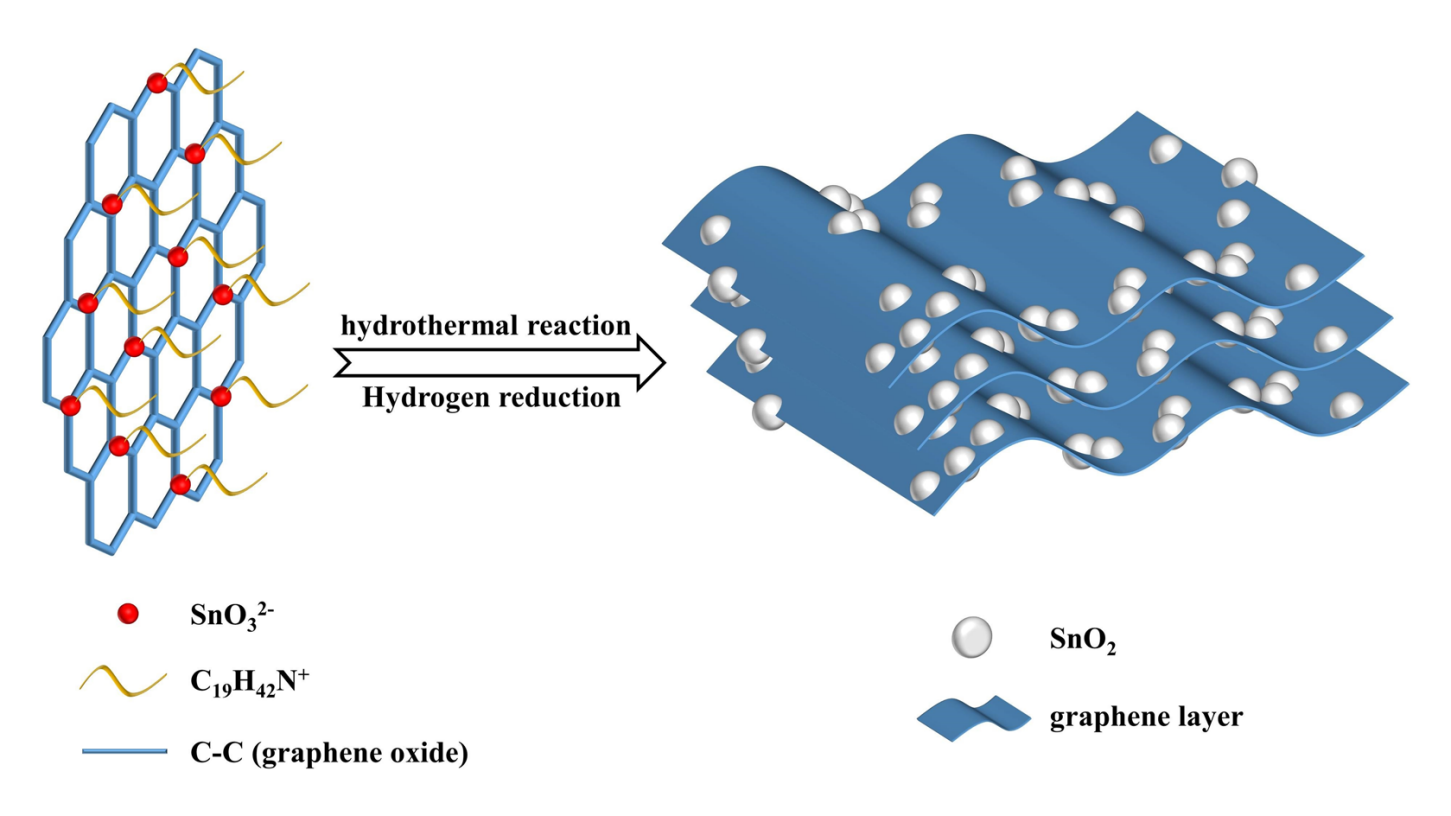
Schematic representation of the synthesis process for SnO_2_/rGO composite.

The tin dioxide/graphene composites in the relevant studies exhibited good cyclic stability owing to the establishment of robust interfacial bonding^15–17^. However, it is necessary to incorporate adequate graphene at least 20 wt.% in the composite. Excessive introduction of graphene not only reduces volumetric specific capacity but also escalates costs. Therefore, our objective is to decrease the graphene in order to optimize cost-effectiveness while preserving the electrochemical properties of the tin dioxide/graphene composite. In this study, SnO_2_-embedded graphene composite with a low graphene content of approximately 5 wt.% was in situ synthesized via a CTAB-assisted hydrothermal-hydrogen reduction route, as shown in Fig. 1. Furthermore, the impact of interfacial modification on the lithium storage performance of SnO_2_/rGO anode was thoroughly investigated.

## Experimental

### Preparation of SnO_2_/rGO materials

All reagents used in this study are of analytical purity grade. First, 0.75 g of sodium stannate (Na_2_SnO_3_·3H_2_O, Aladdin), 2 g of urea (Aladdin), 0.1 g of cetyltrimethylammonium bromide (CTAB, Macklin) and 5 mL of graphene oxide (GO) suspension (dispersed in H_2_O at a concentration of 2 mg/mL, Macklin) were mixed in 35 mL of deionized water, and an additional 40 mL of anhydrous ethanol (Aladdin) was added. The resulting solution was stirred using a magnetic stirrer for 1 h and then transferred into a Teflon-lined stainless-steel autoclave with a volume of 100 mL. Subsequently, the hydrothermal reaction lasted for 24 h at 150 °C in an air-blast drying oven. After naturally cooling to room temperature, the sediment was centrifugally separated and washed with the deionized water. After drying at 70 °C for 10 h in a vacuum oven, the SnO_2_/GO composite was heated at 400 °C in a tube furnace under a reduction atmosphere of hydrogen for 2 h. Finally, SnO_2_/rGO composite was obtained by cooling the samples to room temperature under an inert atmosphere of nitrogen. For comparison, SnO_2_ and SnO_2_/rGO materials without the assistance of CTAB were also synthesized.

### Preparation of anodes

The slurry was prepared by co-grinding the active material, the conductive agent (acetylene black), and the binder (CMC) at a mass ratio of 8:1:1 in deionized water solvent. Subsequently, the slurry was uniformly coated onto the copper foil. Then, the coatings were placed in a vacuum oven at room temperature for 12 h, followed by drying at 110 °C for 3 h. The anode sheets were obtained by cutting the processed copper foil into circular pieces with a diameter of 12 mm.

### Electrochemical measurements

CR2025 coin-type half-cell was assembled using the as-prepared anode in an argon-protected glove box, paring a lithium sheet (Φ16 × 0.4 mm, Trillion Metals) as the counter and reference electrodes. The polypropylene was used as the separator and 1 M LiPF_6_ (the solvent was ethylene carbonate and diethyl carbonate in a volume ratio of 1:1) was used as the electrolyte. Galvanostatic charge–discharge test was performed using a CT2001A battery test system (Land Electronics) at a current density of 1 A g^−1^ at room temperature in a voltage range from 0.01 V to 3.00 V (vs. Li/Li^+^) after activation at a low current density of 0.1 A g^−1^. The rate performance was also evaluated at various current densities (0.1–4 A g^−1^). Furthermore, the pristine cells were employed to carried out electrochemical measurements using a DH7001 electrochemical workstation (Donghua Test). Cyclic voltammetry (CV) test was performed at a scan rate of 0.1 mV/s. Electrochemical impedance spectroscopy (EIS) test was operated over a frequency range of 10^5^ Hz to 10^−2^ Hz, with an applied AC amplitude of 5 mV.

### Structural characterization

Crystal phase of the materials was characterized with a Cu Kα radiation source at a scanning rate of 5° min^-1^, over a 2*θ* angle range of 10° to 80°, using a Bruker D8 Advance X-ray diffraction (XRD) analyzer. The mass fraction of graphene in the composite was analyzed using a TGA 550 thermal analyzer (TA Instruments) with a heating rate of 10 ℃ min^-1^ from room temperature to 800 °C. The chemical states of the elements were determined using an X-ray photoelectron spectrometer (XPS, Thermo Fisher) with an Al Kα radiation source, and the XPS spectra were calibrated according to the C1s peak at 284.8 eV. The particle morphology was observed using a JSM-7900F scanning electron microscope (SEM, Nippon Electronics Co., Ltd), and the distribution of elements in the material was analyzed using its energy dispersive spectrometer (EDS). The crystal microstructure of the anode material was characterized using a Tecnai F30 transmission electron microscope (TEM, FEI).

## Results and discussion

**Figure 2:**
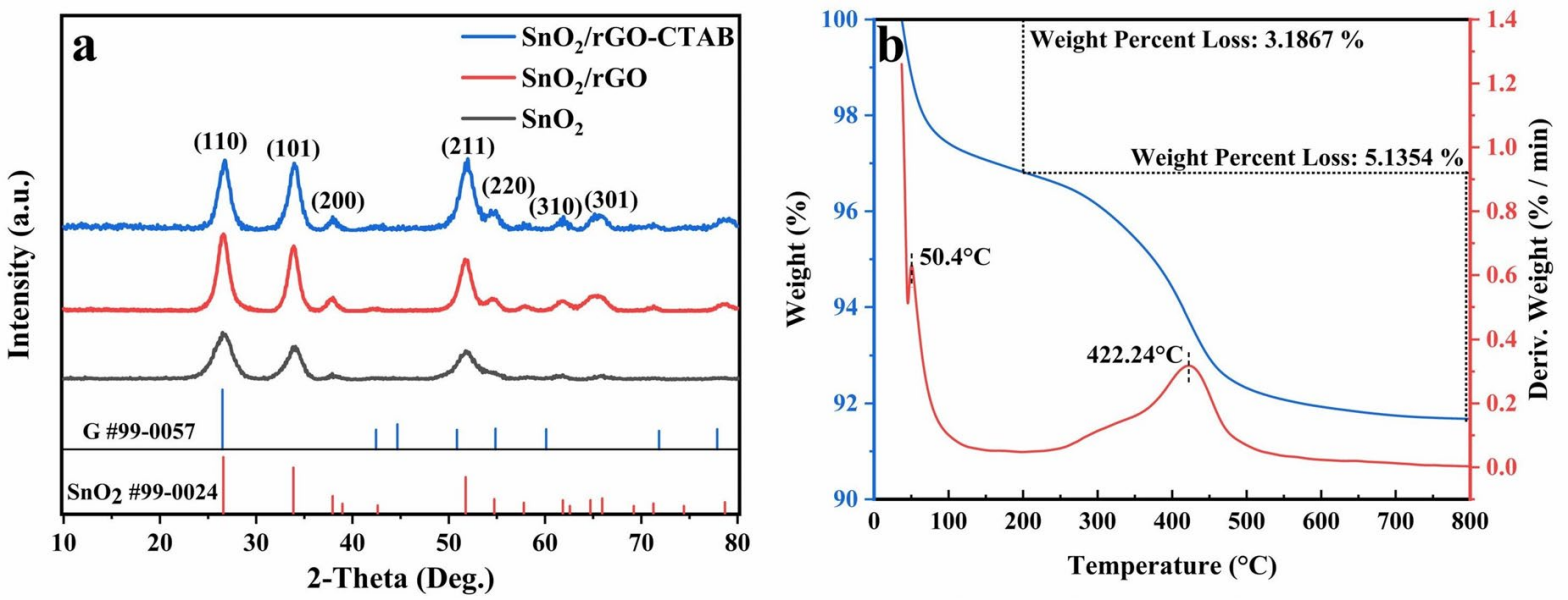
(**a**) XRD patterns of SnO_2_, SnO_2_/rGO and SnO_2_/rGO-CTAB samples; (**b**) TGA curves of SnO_2_/rGO-CTAB sample.

XRD patterns of SnO_2_, SnO_2_/rGO and SnO_2_/rGO-CTAB samples are shown in Fig. 2a. All of the samples exhibit characteristic peaks at 26.6°, 33.9°, and 51.8°, corresponding to the (110), (101), and (211) crystal planes of pure tetragonal SnO_2_ (PDF #99-0024). After the hydrogen reduction, it can be observed that SnO_2_ remains as the predominant component in both SnO_2_/rGO-CTAB and SnO_2_/rGO samples, exhibiting a sharper peak shape. This indicates that SnO_2_ in the sample has not been reduced by hydrogen^18^. However, it is difficult to confirm the presence of graphene in eitherSnO_2_/rGO-CTAB or SnO_2_/rGO sample due to the overlapping peaks around 2*θ* of 26.6° between the (002) crystal plane of graphene and the (110) crystal plane of SnO_2_^19^. TG and DTG curves of SnO_2_/rGO-CTAB are illustrated in Fig. 2b. The minor endothermic peak at 50.4 °C suggests the potential evaporation of adsorbed water in the sample. The weight loss at approximately 422 °C is noteworthy, which can be attributed to the combustion and volatilization of graphene^20^. Thus, the SnO_2_/rGO-CTAB sample contains roughly 5 wt.% of graphene. The results confirm that the composite contains a significantly low weight ratio of graphene.

**Figure 3:**
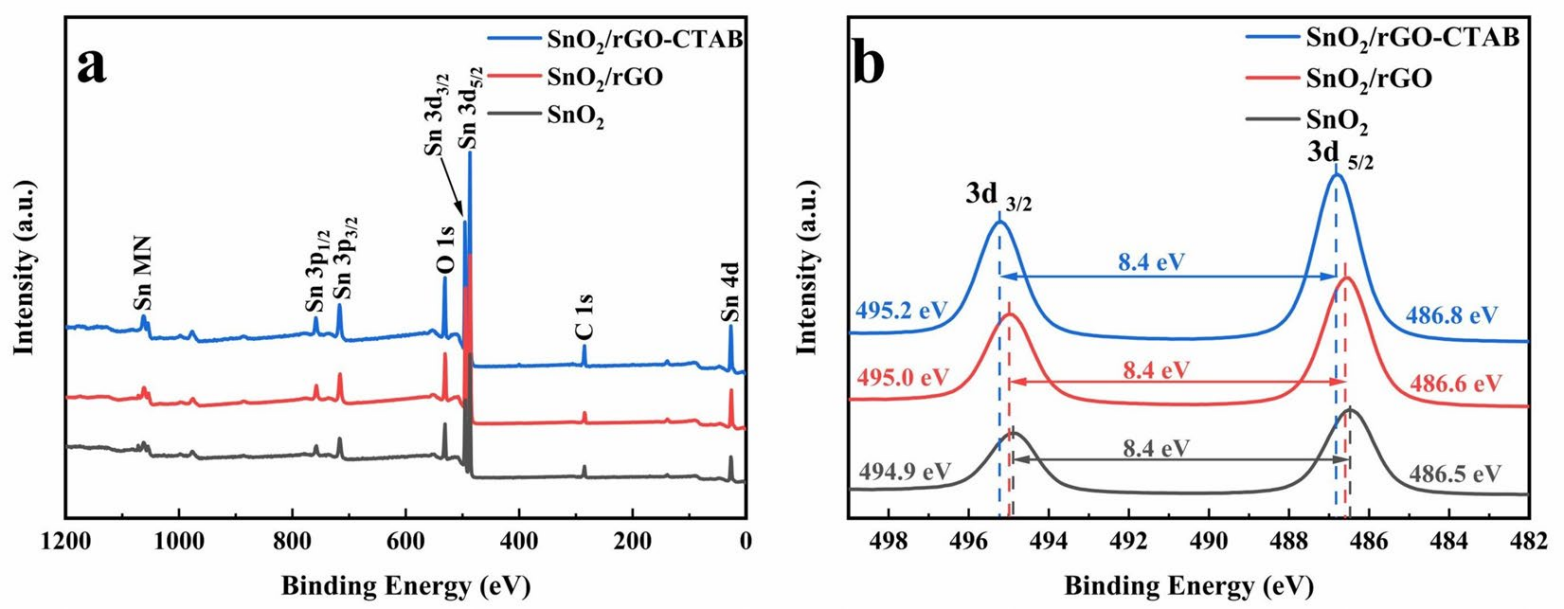
XPS spectra of SnO_2_, SnO_2_/rGO and SnO_2_/rGO-CTAB samples (**a**) survey and (**b**) high-resolution Sn 3d spectra.

XPS spectra of SnO_2_, SnO_2_/rGO and SnO_2_/rGO-CTAB samples are presented in Fig. 3. C, O and Sn elements can be clearly observed from the survey spectra in Fig. 3a. Figure 3b shows the high-resolution Sn 3d spectra of SnO_2_, SnO_2_/rGO and SnO_2_/rGO-CTAB. In the case of the SnO_2_ sample, two distinct peaks at 494.9 eV and 486.5 eV are detected, corresponding to the binding energies of 3d_3/2_ and 3d_5/2_ orbitals for Sn^4+^ respectively. For the SnO_2_/rGO and SnO_2_/rGO-CTAB samples, a binding energy shift implies that the shielding effect on Sn 3d electrons is weakened due to the attraction of graphene to the electron cloud of SnO_2_^15^. The micellar action of CTAB leads to an enhanced SnO_2_/rGO interface, thereby confirming the contribution of CTAB to the construction of Sn–O–C structure.

**Figure 4:**
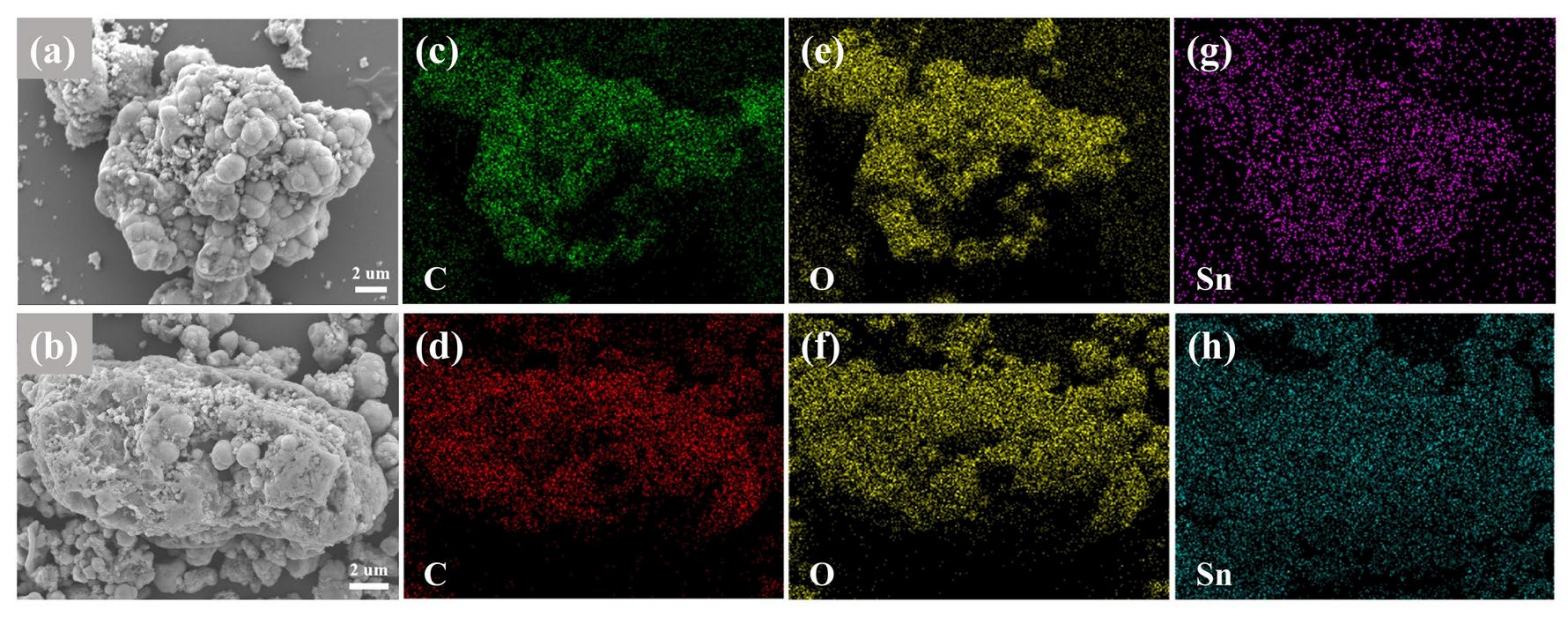
(**a**,**b**) SEM and (**c**–**h**) EDS elemental mapping images of SnO_2_/rGO and SnO_2_/rGO-CTAB samples.

SEM images of the SnO_2_/rGO and SnO_2_/rGO-CTAB samples are displayed in Fig. 4. Figure 4a shows the agglomerated SnO_2_ particles those deposited on the surface of graphene sheets. In Fig. 4b, by contrast, monodispersed SnO_2_ particles are available due to the micellization of CTAB^21^. EDS mapping images (Fig. 4c–h) indicate the uniform elemental distribution of C, O, and Sn. It is reasonable to infer that the inserted SnO_2_ particles can suppress the stacking phenomenon of the graphene interlayers. On the other hand, the graphene can buffer the volume change of SnO_2_ during the cycling process.

**Figure 5:**
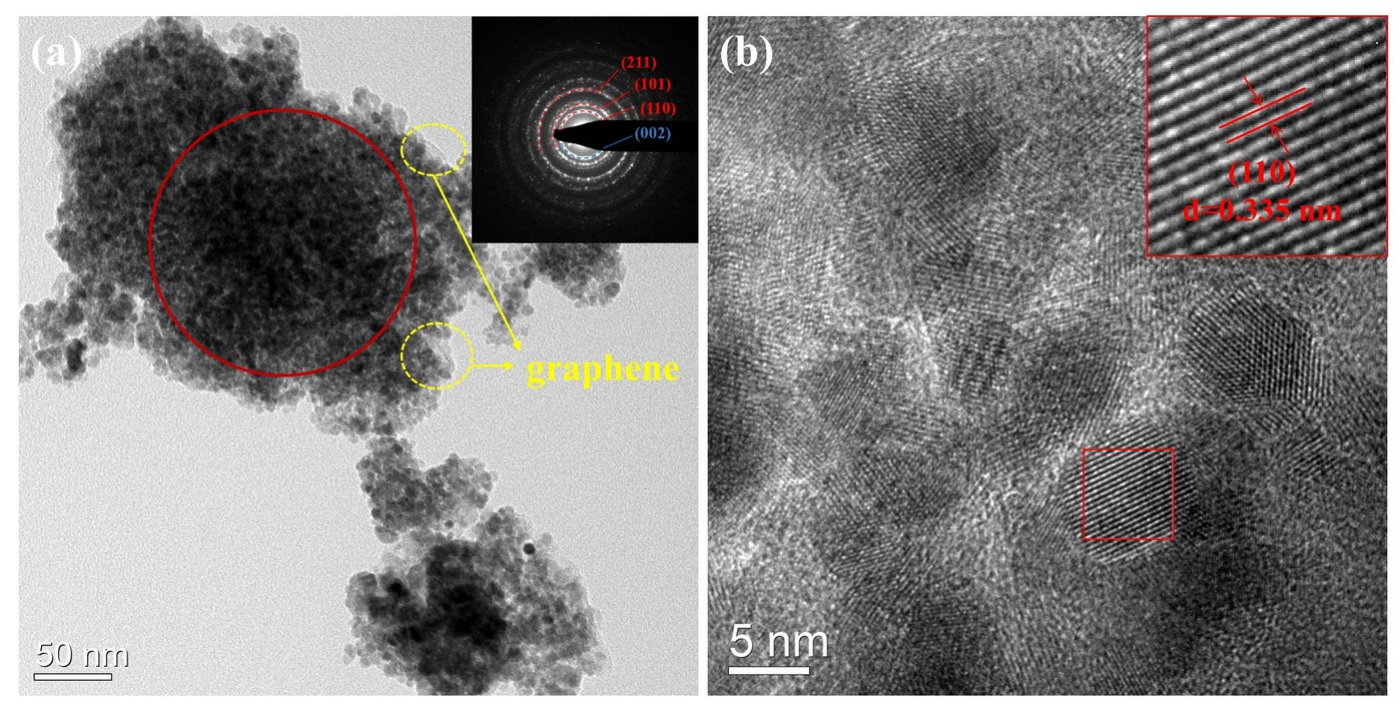
(**a**) TEM image (inset: SAED pattern) and (**b**) HRTEM image (inset: fine structure of (110) crystal plane) of SnO_2_/rGO-CTAB sample.

TEM images in Fig. 5 offer further insight into the morphology and structure of the SnO_2_/rGO-CTAB sample. As shown in Fig. 5a, the SnO_2_ particles are composed of approximately 10 nm grains. Meanwhile, exposed graphene can be seen around the edges of the material. The (110), (101), and (211) crystal planes of SnO_2_, as well as the (002) crystal plane of graphene are calibrated in the pattern of selected area electron diffraction (SAED), which is consistent with the XRD results. From the HRTEM image in Fig. 5b, it can be seen that the sample has a lattice spacing of 0.335 nm, which corresponds to the (110) plane of the tin dioxide crystals, which is in agreement with the SAED results.

**Figure 6:**
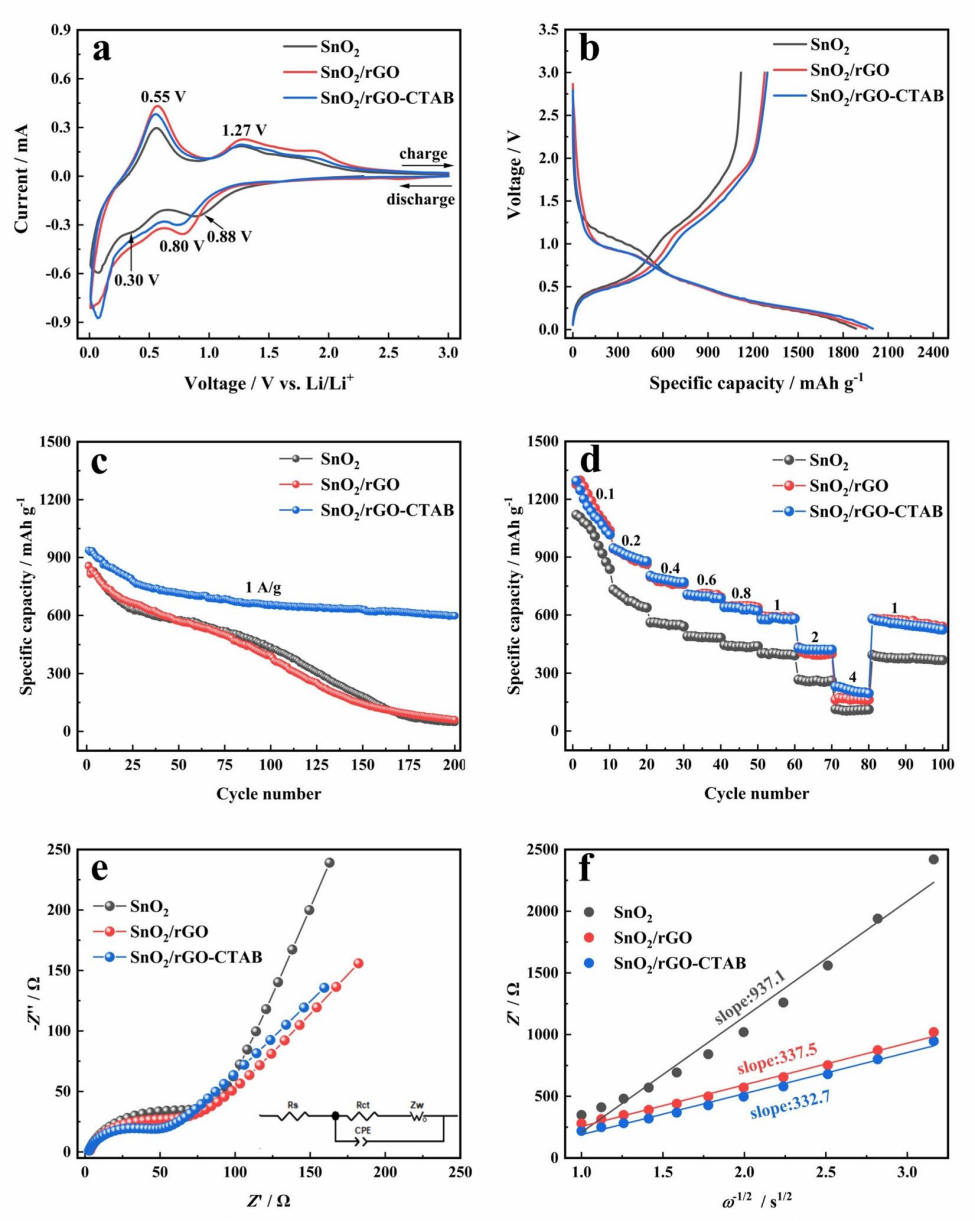
Electrochemical performance of SnO_2_, SnO_2_/rGO, and SnO_2_/rGO-CTAB materials (**a**) CV curves of 1st cycle at 0.1 mV s^−1^, (**b**) Charge–discharge curves of 1st cycle at 0.1 A g^−1^, (**c**) Cycle performance curves at 1 A g^−1^, (**d**) Rate performance curves at 0.1-4 A g^−1^, (**e**) Nyquist plots and equivalent circuit model before cycling, and (**f**) fitting lines for $Z^/$ versus *ω*^*−*1/2^ before cycling.

Cyclic voltammograms of SnO_2_, SnO_2_/rGO, and SnO_2_/rGO-CTAB materials during the first cycle in Fig. 6a reveal the lithiation and delithiation mechanisms. For the pristine SnO_2_, the reduction peak at 0.88 V is assigned to the transformation into Sn and Li_2_O. This peak shifts to 0.8 V for the SnO_2_/rGO and SnO_2_/rGO-CTAB samples because the forming process of solid electrolyte interphase (SEI) on the graphene is involved. Oxidation/reduction peaks appear at 0.55/0.3 V corresponding to the reversible conversion of Li_*x*_Sn (0≦*x*≦4.4) alloy. In addition, the reduction peak around 0.1 V is related to the further alloying reaction between Sn metal and Li^+^. Meanwhile, the more obvious reduction peaks of the SnO_2_/rGO and SnO_2_/rGO-CTAB materials at around 0.1 V are associated with the lithium intercalation into the graphene^22^. These peak potentials are in accordance with the charge and discharge plateaux of the first cycle in Fig. 6b. The ICEs of the SnO_2_, SnO_2_/rGO and SnO_2_/rGO-CTAB materials are determined to be 59.4%, 65.2% and 64.8% respectively. As well known, the ICE of graphene is much higher than that of SnO_2_. Consequently, the SnO_2_/rGO and SnO_2_/rGO-CTAB samples exhibit significantly higher ICEs compared to pure SnO_2_.

Figure 6c presents the cycle performance of the anode materials at a current density of 1 A g^−1^. The specific capacities of SnO_2_ and SnO_2_/rGO samples gradually decrease to 437 mAh g^−1^ and 393 mAh g^−1^ after 100 cycles, and then dramatically declines with only about 50 mAh g^−1^ remaining after 200 cycles. The support of graphene does not effectively improve the structural stability of tin dioxide due to the insufficient adhesive force. In contrast, the interlayer embedded structure formed by the connection of CTAB can buffer the strain induced by volume change and inhibit the agglomeration of SnO_2_ particles^23^. As a result, SnO_2_/rGO-CTAB sample exhibits impressive cyclic stability with a reversible specific capacity of 598 mAh g^−1^ and a capacity retention rate of 63.8% after 200 cycles. Figure 6d shows the rate capabilities of the anode materials in a current density range of 0.1–4 A g^−1^. The capacities of the samples rapidly decrease in the early cycles due to the instable SEI, causing an inevitable consumption of active lithium. At low current densities, the capacity of SnO_2_/rGO sample is comparable to that of SnO_2_/rGO-CTAB sample. However, at a high current density of 4 A g^−1^, SnO_2_/rGO-CTAB sample demonstrates a higher capacity of 231 mAh g^−1^ at 4 A g^−1^ due to its continuous transport channels for ions and electrons, while maintains a reversible capacity of 581 mAh g^−1^ even when restoring the current density to 1 A g^−1^. As shown in Table 1, the initial discharge capacity, cycle and rate performance at high current density of the SnO_2_/rGO-CTAB composite are comparable to state-of-the-art SnO_2_/graphene anode materials. Moreover, the relatively low graphene content of approximately 5 wt.% is in favor of cost reduction which is significant for the commercial application of SnO_2_-based anode materials.

**Table 1:** Comparison of electrochemical performance of various SnO_2_/graphene anode materials for lithium-ion batteries.

Sample	1st discharge capacity (mAh g^−1^)	ICE (%)	Cycle performance	Rate capability	Graphene content (wt.%)
A-SnO_2_/rGO^2,4^	1871	63.5	414 mAh g^−1^ after 300 cycles at 1 C	262 mAh g^−1^ at 5 C	25.8
SnO_2_/Graphene^25^	1762	68.0	1140 mAh g^−1^ after 120 cycles at 0.1 A g^−1^	775 mAh g^−1^ at 2 A g^−1^	22.0
RGO-SnO_*x*_^26^	1307	71.7	767 mAh g^−1^ after 100 cycles at 0.1 A g^−1^	244 mAh g^−1^ at 2 C	19.1
Co-SnO_2_@GS^27^	2103	60.9	828.5 mAh g^−1^ after 200 cycles at 0.5 A g^−1^	675 mAh g^−1^ at 1 A g^−1^	33.2
CMS@SnO_2_/GR^16^	1146	68.9	541.7 mAh g^−1^ after 350 cycles at 0.2 A g^−1^	242 mAh g^−1^ at 2 A g^−1^	38.2
SnO_2_@N-rGO^28^	1904	71.9	1013 mAh g^−1^ after 100 cycles at 0.1 A g^−1^	601 mAh g^−1^ at 2 A g^−1^	8.2
SnO_2_/rGO-CTAB (This work)	1998	64.8	598 mAh g^−1^ after 200 cycles at 1 A g^−1^	431 mAh g^−1^ at 2 A g^−1^	5.3

Figure 6e,f present the Nyquist diagrams of SnO_2_, SnO_2_/rGO, and SnO_2_/rGO-CTAB materials before cycling. The impedance spectra were fitted according to the equivalent circuit (inset in Fig. 6e), using the ZView software. Table 2 shows the corresponding *R*_s_ (the bulk series resistance of the electrolyte) and *R*_ct_ (the charge transfer resistance between electrolyte and electrode materials) for each of the three samples^9^. The *R*_ct_ of SnO_2_, SnO_2_/rGO, and SnO_2_/rGO-CTAB are 61.52 Ω, 37.39 Ω and 34.54 Ω, respectively. The lowest *R*_ct_ of SnO_2_/rGO-CTAB composite benefits from the interlayer embedded structure which results in the abundant active sites and transport pathways for the lithium ions. In addition, the highly dispersed particles are easily infiltrated by the electrolyte and thereby enhancing the interfacial charge transfer. Figure 6f shows the fitted lines for $Z^{/}$ versus *ω*^−1/2^ according to Eq. (1):

1 $${\text{Z}}^{\prime } = {\text{ R}}_{{\text{s}}} + {\text{ R}}_{{{\text{ct}}}} + \sigma \omega^{ - 1/2} ,$$

where $Z^/$ is the real part of the complex impedance, *σ* is the Warburg constant, and *ω* is the angular frequency. The lithium-ion diffusion coefficient of the electrode can be calculated from the *σ* according to Eq. (2):

2 $${\text{D}}_{{\text{Li}}^{+}}=\frac{{\text{R}}^{2}{{\text{T}}}^{2}}{{2}{\text{A}}^{2}{{\text{n}}}^{4}{{\text{F}}}^{4}{{\text{C}}}^{2}{\sigma }^{2}},$$

where *R* represents the gas constant,* T* is the absolute temperature,* A* is the surface area of the cathode, *n* is the number of electrons involved in the reaction, *F* is the Faraday constant, *C* the concentration of lithium-ion, and *σ* the Warburg factor associated with *Z*_W_ (Warburg impedance of lithium-ion diffusion)^22^. The mean *D*_Li+_ values of SnO_2_, SnO_2_/rGO, and SnO_2_/rGO-CTAB are 8.4 × 10^–17^ cm^2^ s^−1^, 6.5 × 10^–16^ cm^2^ s^−1^ and 6.7 × 10^–16^ cm^2^ s^−1^, respectively. The results show that the interlayer embedded structure can shorten the diffusion distance of lithium ions and achieve rapid electron transfer through the Sn–O–C bonds on the surface of the material.

**Table 2:** Impedance parameters of different samples.

Sample	*R*_s_/Ω	*R*_ct_/Ω
SnO_2_	2.58	61.52
SnO_2_/rGO	2.96	37.39
SnO_2_/rGO-CTAB	2.36	34.54

## Conclusion

In this study, SnO_2_/rGO composite is synthesized through a CTAB-assisted hydrothermal-hydrogen reduction route. The micellar action of CTAB facilitates the dispersion of SnO_2_ particles even at a low graphene content of approximately 5 wt.%, and significantly strengthens the bonding between SnO_2_ particles and grapheme matrix. The as-formed interlayer embedded structure not only promotes the transfer of charge carriers, but also buffers the volume change of SnO_2_. Therefore, the SnO_2_/rGO composite exhibits superior cyclic stability that a reversible capacity of 598 mAh g^−1^ and a capacity retention of 63.8% are obtained at a current density of 1 A g^−1^ after 200 cycles, showing a promising prospect as the anode material for high performance lithium-ion batteries.

## Data Availability

Data is provided within the manuscript or supplementary information files.
